# Supplementary material for: Genome-wide association study and pathway analysis identify NTRK2 as a novel candidate gene for litter size in sheep
Source: PLoS One. 2021 Jan 22;16(1):e0244408. doi: 10.1371/journal.pone.0244408 (PMC7822323; doi:10.1371/journal.pone.0244408)
Supplement: Attachment — Submitted filename: PONE-D-20-25223_reviewer comments.docx [file pone.0244408.s004.docx]

**Reviewer Recommendation and Comments for Manuscript Number PONE-D-20-25223**

- The manuscript is acceptable to be published in PLoS One with the below given modifications and additions.

Major comments:

1) The manuscript is well written and researched. Specifically, I would appreciate the authors for trying find out the genes responsible for litter size in sheep but the information provided in the manuscript and the conclusions drawn are too ambitious. There is a need for supporting tests and data, for the drawn conclusions. The conclusions need evidences through minimal wet lab experiments. The supportive data is missing.

2) The author should cite and include more data regarding NTRK2 to support their study.

Minor comments:

1. Please provide justification for the tests used in materials and methods section. Proper justification through citation is missing.
2. As it is mentioned (page 3, lines-54-55) that a previous study by Gholizadeh et al. (2014) identified two significant SNP (rs407696726 and rs412433416) on OAR10 and OAR15 in association with litter size, and in conclusion (page 15, lines-311-312) authors also claimed that they also found a one genomic region on OAR10 associated with the litter size in sheep, how this study is different or novel from the Gholizadeh et al. study?
3. Page 11, lines- 225-226, the citation number given in fig 4 doesn’t match with the reference list. Correction required.

4. Manuscript can be improved a lot in terms of content to be acceptable in Plos one.
